# *PDCD1* and *IFNL4* genetic variants and risk of developing hepatitis C virus‐related diseases

**DOI:** 10.1111/liv.14667

**Published:** 2020-12-29

**Authors:** Valli De Re, Maria Lina Tornesello, Mariangela De Zorzi, Laura Caggiari, Francesca Pezzuto, Patrizia Leone, Vito Racanelli, Gianfranco Lauletta, Stefania Zanussi, Ombretta Repetto, Laura Gragnani, Francesca Maria Rossi, Riccardo Dolcetti, Anna Linda Zignego, Franco M. Buonaguro, Agostino Steffan

**Affiliations:** ^1^ Immunopathology and Cancer Biomarkers/Bioproteomic facility Department of Translational Research Centro di Riferimento Oncologico (CRO) IRCCS Cancer Institute Aviano Italy; ^2^ Molecular biology viral oncology Istituto Nazionale Tumori IRCCS "Fondazione G. Pascale" Napoli Italy; ^3^ Biomedical Sciences and Human Oncology University of Bari Medical School Bari Italy; ^4^ Center for Systemic Manifestations of Hepatitis Viruses (MaSVE) Internal Medicine and Liver Unit Department of Experimental and Clinical Medicine Careggi University Hospital, Florence, Italy Florence Italy; ^5^ Clinical and Experimental Onco‐Hematology Unit Centro di Riferimento Oncologico (CRO) IRCCS Aviano (PN) Italy; ^6^ The University of Queensland Diamantina Institute Translational Research Institute Brisbane Australia

**Keywords:** cryoglobulinaemia, hepatitis virus C, hepatocellular carcinoma, IFNλ4, PD‐1

## Abstract

**Background:**

Genetic variants of *IFNL4* and *PDCD1* genes have been shown to influence the spontaneous clearance of hepatitis C virus (HCV) infection. We investigated the *IFNL4* rs12979860 and the *PDCD1* polymorphisms in 734 HCV‐positive patients, including 461 cases with liver disease of varying severity and 273 patients with lymphoproliferative disorders to determine the association of these genes with patient's outcome.

**Methods:**

Expression levels of *PDCD1* mRNA encoded by haplotypes were investigated by quantitative PCR in hepatocellular carcinoma (HCC) tissue and peripheral blood mononuclear cells. Flow cytometry was used to detect PD‐1 and its ligand PD‐L1.

**Results:**

The frequency of *IFNL4* rs12979860 C/T or T/T genotypes was significantly higher in patients with HCV‐related diseases than blood donors (*P* < .0001). Patients expressing the IFNλ4 variant with one amino acid change that reduces IFNλ4 secretion was found increased in frequency in HCV‐related diseases compared to HCC *PDCD1* mRNA levels in HCC tissue were significantly higher in cases carrying the PD‐1.3 A or the PD‐1.7 G allele (*P* = .0025 and *P* = .0167). Linkage disequilibrium (LD) between PD‐1.3 and IFNL4 was found in patients with mixed cryoglobulinaemia (MC) only (LD = 0 in HCC; LD = 72 in MC). PBMCs of MC patients expressed low levels of PD‐L1 in CD19+IgM+B cells and of PD‐1 in CD4+T cells suggesting the involvement of regulatory B cell‐T cell interaction to the pathogenesis of MC.

**Conclusion:**

Collectively, our data indicate an important contribution of IFNλ4 expression to the development of HCV‐related HCC and an epistatic contribution of *IFNL4* and *PDCD1* in MC.

**Lay summary:**

Studies of *IFNL4* and *PDCD1* genes are helpful to better understand the role of host genetic factors and immune antigens influencing the outcome of HCV‐related diseases. Our data support an association between the expression of IFNλ4, which prevents the expression of IFNλ3, with all the different HCV‐related diseases studied, and besides, evidence that a higher IFNλ4 expression is associated with hepatocellular at a younger age. The expression pattern of low PD‐L1 on B cells and high PD‐1 on CD4+T‐cells in patients with HCV‐positive cryoglobulinaemia suggests a critical role of the PD‐1/PD‐L1 signaling in modulating B cell‐T cell interaction in this lymphoproliferative disease.

**Abbreviations**
BD: blood donors
B‐NHL: B‐cell non‐Hodgkin lymphoma
CI: confidence interval
HCC: hepatocellular carcinoma
HCV: hepatitis C virus
MAF: minor allele frequency
MC: mixed cryoglobulinaemia
OR: odds ratio
PD‐1: programmed cell death protein 1
SNP: single nucleotide polymorphisms

## 1 INTRODUCTION

Individuals with a hepatitis C virus (HCV) infection develop chronic hepatitis C (CHC) or remain asymptomatic. Recently, the incidence of hepatocellular carcinoma (HCC) has increased in association with the increase in HCV infection.^1^ Chronic viral infection is characterized by dysfunctional B cells, which render some patients susceptible to the development of B lymphocyte proliferative diseases including mixed cryoglobulinaemia (MC) and B‐cell non‐Hodgkin lymphoma (B‐NHL).^2^, ^3^ Interferon‐lambda 4 (*IFNL4)* and programmed cell death protein‐1 (PD‐1) expression levels have emerged as important modulators of altered host response to HCV infection ^4^, ^5^, ^6^, ^7^ and as cause of a variety of autoimmune diseases.^8^ Immunotherapies that inhibit the interaction between PD‐1 and its ligands PD‐L1 and PD‐L2 have been shown to confer a substantial survival benefit in patients with HCC^9^, ^10^ as well as in those with haematological^11^ or infectious diseases.^12^ The *IFNL4* gene encodes the IFNλ4 protein that increases the expression of IFN‐stimulated genes (ISGs) in the liver. The receptors for IFNλ4 are expressed on selective cell types like hepatocytes and some immune cells.^13^ IFNλ4 is expressed only in individuals carrying the *IFNL4* rs368234815 ∆G allele, which creates an open reading frame. In contrast, individuals with the rs368234815 T/T allele do not express the protein because of a premature stop codon. Other *IFNL4* polymorphisms include a common variant (rs117648444) resulting in a P70S amino acid change and two others (L79F and K154E), which lead to lower IFNλ4 secretion and antiviral activity.^14^, ^15^ High IFNλ4 expression is strongly associated with spontaneous HCV clearance and a lower response to IFN‐based treatment.^16^, ^17^ The *IFNL4* rs12979860[T] allele variant may be used as an alternative to *IFNL4* rs368234815 ΔG variant to test for *IFNL4* expression since their almost complete linkage is well established.^18^ IFNλ4 was recently found to have selective pressure on the HCV genome.^19^ Genome‐wide association studies of patients with hepatitis infection identified polymorphisms in *IFNL4* and *PDCD1* genes conferring a higher risk of chronic infection, HCV‐related diseases and treatment response.^8^, ^20^, ^21^ Data from patients with HCV‐related lymphoproliferative diseases are limited, and there is a clear need to verify the role of IFNλ4 in the Caucasian population. The rs11568821 (PD‐1.3) was associated with the development of autoimmune diseases in Europeans and Mexicans but not in African Americans.^22^ In addition, the incidence of HCC is higher among Asian Americans than White Americans^23^, while a high incidence of HCV‐related MC was present in the Mediterranean basin,^24^ suggesting that genetic polymorphisms and environmental risk factors may influence the risk of these diseases. Investigation of *IFNL4* and *PDCD1* immunogenetic profiles in patients with different HCV‐related diseases could help to elucidate these pathologies. We therefore studied *IFNL4* and *PDCD1* genetic variants distribution and their relative expression in Italian patients diagnosed with different HCV‐related diseases. Results could underlie the complex immune interactions leading to different clinical outcomes in chronic HCV‐positive patients.

## 2 PATIENTS AND METHODS

This study investigated data and biological samples obtained from 734 HCV‐positive patients with different liver or lymphoproliferative diseases and 94 non‐HCV‐infected blood donors. Liver diseases taken into consideration were CHC (n = 148), cirrhosis (n = 113) and HCC (n = 200), while the studied lymphoproliferative diseases were MC (n = 138) and NHL (n = 135). Patients were in treatment at one of the three participating Italian hospitals where they had been diagnosed and had undergone HCV molecular analysis. For each patient, we obtained from clinical records the following data: age at diagnosis, sex, HCV positivity, HCV viral load and genotype, and specific diagnosis. Data on blood donors’ age and sex were collected.

In agreement with the Centre for Disease Controls and Prevention (CDC) guidelines, we defined as CHC a patient with long‐lasting HCV infection who had persistent viraemia for more than 6 months after initial exposure diagnosed by blood test. Such test is based on the detection of serum anti‐HCV antibodies performed by an enzyme immunoassay (III‐generation EIA) against antigens from the HCV‐core and from the HCV‐nonstructural regions and is confirmed by the qualitative analysis that detects the presence of HCV RNA (Cobas Amplicor HCV assay). All patients we have included in the CHC group were positive to both these tests. The viral load was determined in 192 patients (Table 1) to monitor their response to treatment. Data reported in Table 1 were referred to tests performed before treatment. The viral load (branched DNA, Chiron, Emeryville, USA) was measured only in a subgroup of patients with HCV infection because in most cases a positive or a negative result was sufficient for clinical determinations. Sustained virological response (SVR) is defined as a negative response to serum HCV RNA viral load performed at least 12 weeks after the end of HCV treatment.

**TABLE 1:** Baseline characteristics of HCV patients and blood donors

Characteristics									
Age (years)	BD CHC Cirrhosis HCC MC NHL	42 ± 10 57 ± 14 64 ± 11 67 ± 9 66 ± 10 68 ± 15							
Sex male	BD CHC Cirrhosis HCC MC NHL	90.1% 48.3% 65.4% 71.2% 29.3% 47.7%							
HCV positive viraemia^a^	BD CHC^b^ Cirrhosis HCC MC NHL	0% 100% 100% 100% 100% 100%							
HCV viral load^b^	CHC Cirrhosis HCC MC NHL	2.5 ± 0.3 2.2 ± 0.4 1.9 ± 0.2 3.1 ± 0.2 3.0 ± 0.4							
HCV genotype^c^	CHC Cirrhosis HCC MC NHL	genotype 1	60% 63% 58% 63% 60%	genotype 2	26% 21% 28% 26% 32%	genotype 3	13% 10% 9% 7% 4%	genotype 4	2% 6% 4% 4% 4%
CHC with									
Mild‐moderate fibrosis Mild‐moderate fibrosis	60	40.5%							
Severe fibrosis	59	39.9%							
Data not available	29	19.6%							
MC with									
Mild‐moderate fibrosis	27	19.6%							
Severe fibrosis	24	17.4%							
Data not available	87	75.0%							

Note

Continuous variables reported as mean ± standard deviation. Categorical variables reported as %.

BD, blood donors, CHC, patients with a chronic HCV infection for more than 6 months; HCC, patients with hepatocellular carcinoma; MC, patients with mixed cryoglobulinaemia; NHL, patients with a non‐Hodgkin lymphoma.

^a^ HCV infection for more than 6 months confirmed at both serum anti‐HCV antibodies and for the presence of HCV RNA.

^b^ Data available for 192 cases;

^c^ Data available for 323 cases.

In addition, fibrosis stage assessment by liver biopsy (Metavir score) or fibroscan measure in patients with CHC and MC were retrieved when available (n = 119 CHC; n = 51 MC, Table 1). A good correlation was established between fibroscan and Metavir score as follow: F0‐F1, 7.1 kPa; F2, 7.1‐9.4 kPa; F3, 9.5‐12.5 kPa; and F4, 12.5 kPa. We categorized patients with F0‐F2 stage as a 'mild‐moderate fibrosis' and those with a F3‐F4 stage as 'advanced fibrosis'.

HCV genotypes have been determined using the Versant HCV Genotype 2.0 assay (Siemens Healthcare Diagnostics). A diagnosis of HCC was based on the criteria of the European Association for the Study of the Liver^25^ using non‐invasive criteria, namely two imaging techniques both demonstrating a focal lesion >2 cm in diameter with features of arterial hypervascularization. The diagnosis of cyroglobulinaemia was based on the detection of cryoglobulins, performed according to guidelines of the Associazione Italiana per la Lotta alle Crioglobulinemie. The diagnosis of NHL in the course of HCV infection has been histopathologically confirmed based on the WHO classification.^26^

The study protocol was approved by the Comitato Etico Indipendente of the Azienda Ospedaliero‐Universitaria Consorziale Policlinico di Bari, the Scientific Board and Ethics Committee of Fondazione G. Pascale Istituto Nazionale Tumori, the institutional review board code SPE 14.084 AOUC, Comitato Etico Area Vasta Centro AOU Careggi, Firenze and Comitato Etico Bio‐banca CRO. The study was done in accordance with the Declaration of Helsinki. All research subjects provided written informed consent for the collection, storage and analysis of their samples and data. The analyses were carried out at the Centro di Riferimento Oncologico (Aviano, Italy) and Fondazione G. Pascale (Naples, Italy) in the period 2017‐2019.

### 2.1 Biological samples and nucleic acid extraction

At first diagnosis, patients had contributed a sample of venous blood for research purposes. Specifically, 461 patients were diagnosed with increasing severity of liver disease, from CHC towards cirrhosis and HCC, and 273 patients with MC or NHL (Table 1). Whole blood (2 mL) was obtained from each patient and cryopreserved at −20°C until use. Total genomic DNA was extracted from whole blood using DNeasy Kits (Qiagen). From most HCC patients, peripheral blood mononuclear cells (PBMCs) had been isolated (from 10 mL blood) by Ficoll‐Hypaque density gradient centrifugation. HCC patients had also contributed part of a biopsy sample of liver tissue that was not needed for pathological examination; this part had been stored in RNAlater stabilizing solution at −80°C until use. Total DNA was extracted from HCC biopsies according to published procedures.^27^ Total RNA was extracted from approximately 20 mg of frozen HCC tissue and approximately 1 × 10^6^ PBMCs using Trizol Reagent following manufacturer's instructions. Quality and quantity of isolated nucleic acids were assessed with the spectrophotometer Nanodrop 2000C (Thermo Fisher Scientific). Samples with ratio of absorbance at 260 nm and 280 nm equal to or above 1.8 were further analysed.

### 2.2 Selection of known IFNL4 and PDCD1 polymorphisms and IFNL4 mutational analysis

For *IFNL4,* we chose to genotype rs12979860 because of its known linkage disequilibrium with rs368234815 which is required for IFNλ4 expression^4^ and because we had validated a method for rs12979860 genotyping in a previous study.^17^

For *PDCD1*, we selected single nucleotide polymorphisms (SNPs) known to be associated with susceptibility to cancer or autoimmune diseases^28^, ^29^, ^30^, ^31^, ^32^, ^33^, ^34^, ^35^, ^36^, ^37^, ^38^, ^39^, ^40^, ^41^, ^42^, ^43^, ^44^, ^45^ or to influence the spontaneous resolution of HCV infection or the response to antiviral treatment^46^, ^47^, ^48^, ^49^ (Table S1). From this preliminary list of six SNPs, we selected four SNPs (ie PD‐1.3, PD‐1.5, PD‐1.6 and PD‐1.7) for subsequent analysis because they had a minor allele frequency (MAF) ≥0.05 in the Italian population according to the Ensemble website database (http://www.ensembl.org/Homo_sapiens/; Table S1).

To identify other genetic variations potentially affecting IFNλ4 activity, we sequenced the *IFNL4* gene (intron 1 to exon 5) in genomic DNA from PBMCs of 36 cases homozygous at rs12979860 (16 C/C and 20 T/T). Sequencing was done as previously described^4^ but with a modified sequencing primer (IFNL4 int IV1, Table S2). The amino acid changes G58R and P70S in the IFNλ4 protein flank residue N61, whose glycosylation is required for secretion of active IFNλ4 protein.^50^ Consequently, these variants lower the antiviral activity of the reference IFNλ4.^51^

### 2.3 Genotyping and sequencing of IFNL4 and PDCD1

Genotyping of *IFNL4* rs12979860 and rs117648444 (C>T, P70S) was performed using custom TaqMan SNP genotyping assays (Applied Biosystems) on a 7900HT Fast Real‐Time PCR system (Applied Biosystems).

To sequence *IFNL4* (from intron 1 to exon 5), we first amplified the gene from genomic DNA by PCR in a reaction volume of 25 μl (200 ng dNTPs and 0.5 U GoTaq DNA Polymerase, Promega) using primers as reported in Table S2 and cycling conditions as described in reference 4. Sequencing was performed as previously reported.^52^

To genotype *PDCD1* SNPs, genomic DNA (30‐300 ng) was amplified in a 50 μL reaction mixture containing 10 pmol of each primer (Table S2), 1.25 U Hot Master Taq DNA Polymerase (5 Prime) and 25 μL PreMixJ (MasterAmp PCR, Epicentre, Madison, USA) on a Sure Cycler 8800 thermal cycler (Agilent Technologies). Amplification started with an initial denaturation at 94°C for 3 min, followed by 30 amplification cycles of denaturation at 94°C for 30 s, annealing at 65°C for 30 s, elongation at 72°C for 1 min, and a 10 min final elongation at 72°C. PCR products were subjected to Sanger automated sequencing analysis.

### 2.4 LD analysis

Linkage disequilibrium (LD) was estimated among PD‐1, that is, PD‐1.3, PD‐1.5, PD‐1.7 and IFNL4 rs12979860 SNP. Distribution of the Lewontin's coefficient D′ and correlation coefficient *r* ^2^ was calculated as the measures of LD using the SHEsis online tool (http://analysis.bio‐x.cn). LD within each genomic region was explored and the extent of statistical significance of each pairwise association was represented by a scale of colour intensity.

### 2.5 Analysis of PDCD1 mRNA expression

The expression levels of haplotypes within *PDCD1* gene were evaluated by real‐time quantitative PCR using total RNA from PBMCs of selected cases based on their *PDCD1* haplotype (n = 12) and from selected HCC tissues (n = 12). Briefly, total RNA (500 g) was reverse transcribed in a 20 μL volume with Superscript II RNase H and an oligo d(T) primer (Life Technologies). The obtained cDNA (2 µL) was then subjected to qPCR using forward (5’‐GAGGGACAATAGGAGCCAGG‐3’) and reverse (5’‐TCTTCTCTCGCCACTGGAAA‐3’) primers targeting *PDCD1* exons 4 and 5, respectively, to exclude the amplification of cross‐contaminating DNA. Each reaction contained 12.5 μL of 1x iQ SYBR Green supermix (Bio‐Rad Laboratories), 10 pmol of each primer, 2 μL of cDNA and nuclease‐free water in a final volume of 25 μL. Amplifications were performed in triplicate using the CFX96 Real‐Time PCR Detection System (Bio‐Rad Laboratories). Gene expression was analysed using the comparative Ct (2^‐ΔCt^) method of quantification and *SRSF4* as reference sample. The ΔCt values for each transcript were calculated by subtracting the respective Ct (cycle threshold) value from the corresponding *SRSF4* Ct (ΔCt = Ct_x_ – Ct*_SRSF4_*). Ct values were corrected for the efficiency of primer pairs.^53^

### 2.6 PD‐1 and PD‐L1 flow cytometry analysis

Cryopreserved peripheral blood mononuclear cells (PBMCs) from two HCV‐negative patients with HCC, two patients with HCV‐related HCC and two patients with HCV‐related MC were used for *PD‐1* and *PD‐L1* flow cytometry analysis. Before the analysis, the dimethyl sulfoxide (DMSO) cryoprotectant was removed by washing and centrifugation. PBMCs were re‐suspended in phosphate‐buffered saline (PBS). Sample were stained with combination of vital dye 7‐aminoactinomycin (7‐AAD), CD4 PE (cl. SK3), PD‐1 FITC (cl.MIH4), or CD19 APC (cl.HIB19), PD‐L1 PE (cl.MIH1), all from Becton Dickinson, San Jose, CA) and IgM FITC (Dako). All samples were acquired on a FACSCantoII flow cytometer and DiVa software (Becton Dickinson). At least 1,000 T or B cells were acquired per tube. Media of percent positive cells from vital lymphocytes were calculated between HCC HCV‐negative, HCC HCV‐positive or MC‐HCV‐positive samples.

### 2.7 Statistical analysis

Fisher's exact test and ANOVA analysis of variance were used to compare allele and genotype frequency of gene polymorphisms between patient groups with different pathologies and control subjects.

Multivariate logistic regression analysis was performed with diagnosis as the dependent variable and genotype as independent variable; age and sex were valuated as covariables. Odds ratios and 95% confidence intervals were calculated. Genotypes of each polymorphism were assessed according to dominant (0 wild‐type homozygote; 1 heterozygote and variant homozygote), recessive (0 wild‐type homozygote and heterozygote; 1 variant homozygote) and additive genetic models (ie overdominant and log‐additive).

The chi‐squared test for trends was used to assess associations with genotype for liver diseases of increasing severity (chronic HCV infection, cirrhosis and HCC).

Statistical analyses were performed using MedCalc software (version 17.2), SNPStats (https://www.snpstats.net/start.htm) and SHEsis online tool (http://analysis.bio‐x.cn). A *P* < .05 was taken to indicate statistical significance. Bonferroni for dependent SNPs and Sidak corrections for independent SNPs were utilized to conduct multiple comparisons test.

## 3 RESULTS

This study examined the immunogenetic profiles of 734 HCV‐infected patients, 461 with a liver disease (148 CHC, 113 cirrhosis, 200 HCC) and 273 with a lymphoproliferative disease (138 MC, 135 NHL) and 98 non‐HCV‐infected blood donors, all living in Italy (Table 1). Among the patients with liver disease, there were 148 with chronic HCV infection, 113 patients with cirrhosis and 200 patients with HCC. Among those with a lymphoproliferative disease, there were 138 with MC and 135 with B‐NHL. Male sex predominated among cirrhosis and HCC patients, while female sex predominated among MC patients. Blood donors (BD) were mostly male. Fibrosis data were available for 119 patients included in the CHC group and for 51 patients included in the MC group (Table 1). Among the CHC group, 60 patients (40.5%) were diagnosed with mild‐moderate liver fibrosis (fibrosis F1‐F2) and 59 (39.9%) with severe fibrosis (F3‐F4); in the MC group, 27 patients (19.6%) had mild‐moderate liver fibrosis and 24 (17.4%) a severe fibrosis (F3‐F4). Response data to anti‐HCV treatment were available for 319 patients: 101 with CHC, 82 with cirrhosis, 34 with HCC, 66 with MC and 36 with NHL.

### 3.1 IFNL4 and PDCD1 genotype frequencies

Patients and controls were genotyped for five SNPs having possible immune‐modulating effects, including rs12979860 in *IFNL4* and four SNPs in *PDCD1* genes (Table S1). The *PDCD1* SNPs, termed PD‐1.3 (rs11568821), PD‐1.5 (rs2227981), PD‐1.6 (rs10204525) and PD‐1.7 (rs7421861), had a MAF ≥0.05 in the reported Toscani population in Italy as well as in BD (Table S1). Genotype frequencies of the five SNPs are given in Table 2, in Table S3 according to fibrosis and in Table S4 according to SVR.

**TABLE 2:** Frequencies of PD‐1 and IFNL4 genotypes in patients with HCV‐related diseases and in blood donors

	PD‐1.3 (%) rs11568821	#	PD‐1.5 (%) rs2227981	#	PD‐1.6 (%) rs10204525	#	PD‐1.7 (%) rs7421861	#	IFNL4 (%) rs12979860	#
BD (n = 98)	G/G 82 (84%) G/A 16 (16%)		C/C 36 (37%) C/T 44 (45%) T/T 18 (18%)		C/C 79 (81%) C/T 19 (19%) T/T 0		A/A 42 (43%) A/G 47 (48%) G/G 9 (9%)		C/C 44 /45%) C/T 48 (49%) T/T 6 (6%)	
CHC (n = 148)	G/G 114 (77%) G/A 34 (23%)		C/C 39 (26%) C/T 85 (57%) T/T 24 (16%)		C/C 125 (84%) C/T 20 (14%) T/T 3 (2%)		A/A 78 (53%) A/G 56 (38%) G/G 14 (9%)		C/C 39 (26%) C/T 87 (59%) T/T 22 (15%)	
A) Compared to BD^a^										
CHC (n = 148)	G/G 114 (77%) G/A 34 (23%)		C/C 39 (26%) C/T 85 (57%) T/T 24 (16%)		C/C 125 (84%) C/T 20 (14%) T/T 3 (2%)		A/A 78 (53%) A/G 56 (38%) G/G 14 (9%)		C/C 39 (26%) C/T 87 (59%) T/T 22 (15%)	log‐additive 2.04 (1.33‐3.13) *P < .0001*
Cirrhosis (n = 113)	G/G 92 (81%) G/A 21 (19%)		C/C 34 (30%) C/T 58 (51%) T/T 21 (19%)		C/C 93 (82%) C/T 19 (17%) T/T 1 (1%)		A/A 53 (47%) A/G 44 (39%) G/G 16 (14%)		C/C 26 (23%) C/T 66 (58%) T/T 21(19%)	**dominant** **2.73 (1.51‐4.93)** ***P < .0001***
HCC (n = 200)	G/G 151 (75%) G/A 45 (23%) A/A 4 (2%)		C/C 74 (37%) C/T 94 (47%) T/T 32 (16%)		C/C 159 (80%) C/T 39 (20%) T/T 2 (1%)		A/A 75 (38%) A/G 114 (57%) G/G 11 (5%)		C/C 51 (26%) C/T 103 (52%) T/T 46 (23%)	**log‐additive** **2.28 (1.55‐3.35)** ***P < .0001***
MC (n = 138)	G/G 99 (72%) G/A 39 (28%)	recessive 2.02 (1.05‐3.87) *P = .030*	C/C 53 (38%) C/T 62 (45%) T/T 23 (17%)		C/C 109 (79%) C/T 28 (20%) T/T 1 (1%)		A/A 55 (40%) A/G 63 (46%) G/G 20 (14%)		C/C 58 (42%) C/T 61 (44%) T/T 19 (14%)	recessive 2.45 (0.94‐6.38) *P = .053*
NHL (n = 135)	G/G 113 (84%) G/A 22 (16%)		C/C 41 (30%) C/T 60 (45%) T/T 34 (25%)		C/C 106 (79%) C/T 27 (20%) T/T 2 (2%)		A/A 69 (51%) A/G 54 (40%) G/G 12 (9%)		C/C 45 (33%) C/T 66 (49%) T/T 24 (18%)	**recessive** **3.28 (1.29‐8.37)** ***P = .0069***
Total liver diseases (n = 461)	G/G 357 (77%) G/A 100 (22%) A/A 4 (1%)		C/C 147 (32%) C/T 237 (51%) T/T 77 (17%)		C/C 377 (82%) C/T 78 (17%) T/T 6 (1%)		A/A 206 (45%) A/G 214 (46%) G/G 41 (9%)		C/C 116 (25%) C/T 256 (56%) T/T 89 (19%)	**log‐additive** **2.23 (1.56‐3.19)** ***P < .0001***
Lymphoproliferative diseases (n = 273)	G/G 212 (78%) G/A 61 (22%)		C/C 94 (34%) C/T 122 (45%) T/T 57 (21%)		C/C 215 (79%) C/T 55 (20%) T/T 3 (1%)		A/A 124 (45%) A/G 117 (43%) G/G 32 (12%)		C/C 103 (38%) C/T 127 (47%) T/T 43 (16%)	**recessive** **2.84 (1.17‐6.89)** ***P = .011***
All HCV‐positive patients (n = 734)	G/G 569 (78%) G/A 161 (22%) A/A 4 (1%)		C/C 241 (33%) C/T 359 (49%) T/T 134 (18%)		C/C 592 (81%) C/T 133 (18%) T/T 9 (1%)	.	A/A 330 (45%) A/G 331 (45%) G/G 73 (10%)		C/C 219 (30%) C/T 383 (52%) T/T 132 (18%)	**log‐additive** **1.86 (1.33‐2.60)** ***P < .0001***
B) Compared to CHC^a^										
Cirrhosis (n = 113)	G/G 92 (81%) G/A 21 (19%)		C/C 34 (30%) C/T 58 (51%) T/T 21 (19%)		C/C 93 (82%) C/T 14 (12%) T/T 6 (5%)		A/A 53 (47%) A/G 44 (39%) G/G 16 (14%)		C/C 26 (23%) C/T 66 (58%) T/T 21(19%)	
HCC (n = 200)	G/G 151 (75%) G/A 45 (22%) A/A 4 (2%)	Recessive *P = .035*	C/C 74 (37%) C/T 94 (47%) T/T 32 (16%)	dominant 0.61 (0.38‐0.97) *P = .035*	C/C 159 (80%) C/T 39 (19%) T/T 2 (1%)		A/A 75 (38%) A/G 114 (57%) G/G 11 (5%)	**dominant** **1.86 (1.21‐2.86) *P < .005***	C/C 51 (26%) C/T 103 (52%) T/T 46 (23%)	
MC (n = 138)	G/G 99 (72%) G/A 39 (28%)		C/C 53 (38%) C/T 62 (45%) T/T 23 (17%)	dominant 0.57 (0.35‐0.95) *P = .029*	C/C 109 (79%) C/T 28 (20%) T/T 1 (1%)		A/A 55 (40%) A/G 63 (46%) G/G 20 (14%)	log‐additive 1.48 (1.04‐2.09) *P = .026*	C/C 58 (42%) C/T 61 (44%) T/T 19 (14%)	**dominant** **0.50 (0.30‐0.82)** ***P = .006***
NHL (n = 135)	G/G 113 (84%) G/A 22 (16%)		C/C 41 (30%) C/T 60 (45%) T/T 34 (25%)	overdominant 0.59 (0.37‐0.95) *P = .029*	C/C 106 (79%) C/T 27 (20%) T/T 2 (2%)		A/A 69 (51%) A/G 54 (40%) G/G 12 (9%)		C/C 45 (33%) C/T 66 (49%) T/T 24 (18%)	
Liver diseases excluding CHC (n = 313)	G/G 243 (78%) G/A 66 (22%) A/A 4 (1%)		C/C 108 (34%) C/T 152 (49%) T/T 53 (17%)		C/C 252 (81%) C/T 58 (18%) T/T 3 (1%)		A/A 128 (41%) A/G 158 (50%) G/G 27 (9%)	dominant 1.61 (1.09‐2.39) *P = .02*	C/C 77 (25%) C/T 169 (54%) T/T 66 (21%)	
Lymphoproliferative diseases excluded CHC (n = 273)	G/G 212 (78%) G/A 61 (21%)		C/C 94 (34%) C/T 122 (45%) T/T 57 (21%)	**overdominant** **0.60 (0.40‐0.90)** ***P = .012***	C/C 215 (79%) C/T 55 (20%) T/T 3 (1%)		A/A 124 (45%) A/G 117 (43%) G/G 32 (12%)		C/C 103 (38%) C/T 127 (47%) T/T 43 (16%)	dominant 0.59 (0.38‐0.92) *P = .017*

Abbreviations: BD, blood donors; CHC, chronic HCV infection; HCC, hepatocellular carcinoma; MC, autoimmune lymphoproliferative mixed cryoglobulinaemia; NHL, non‐Hodgkin lymphoma.

^a^ The most significant model for association with diagnosis (OR (95% CI) in comparison with BD is reported only when *P* value is significant (*P* < .05). A Bonferroni's correction for multiple SNPs results in a P‐value threshold of 0.0125 and of Sidak's correction of 0.0127. Significant SNPs after corrections are in bold text.

Multivariate logistic regression was done to identify the associations between genotype and clinical group according to different genetic models (Table 2). The *IFNL4* rs12979860[T] allele associated with HCV positivity (n = 734) compared to BD (n = 98), in a log‐additive model with an odds ratio (OR) of 1.86 (95% CI, 1.33‐2.60, *P* < .0001), that remains significant after both Bonferroni's and Sidak's corrections. The association was more evident in patients with liver diseases, who had a lower frequency of the protective genotype C/C than patients with a lymphoproliferative disease (25% vs 38%; Table 2). T/T genotype showed a positive trend with the increase in liver disease severity (*P* = .018. Table S5) and apparently not affected by a sustained response to anti‐HCV treatment (Table S6).

The four *PDCD1* SNPs showed no association when genotype frequencies were compared between all HCV‐positive patients and BD (Table 2). The analysis of individual clinical groups showed a higher frequency of PD‐1.3 G/A genotype only among MC patients compared to BD (28% vs 16%); in a recessive model, OR = 2.02 (95% CI, 1.05‐3.87; *P* = .030). These results suggest that *PDCD1* polymorphisms have no effect on the clearance of HCV infection. Further examination revealed associations between *PDCD1* SNP genotypes and type of liver disease. In particular, patients with HCC had a higher frequency of PD‐1.5 C/C alleles (37% vs 26%, OR = 0.61, *P* = .035) and a lower frequency of PD‐1.7 A/A (53% vs 38%, OR = 1.86, *P* < .005) compared to those with CHC. After Bonferroni's and Sidak’ s correction only PD‐1.7 A/A association remained significant. The PD‐1.7 A/A allele distribution in liver diseases has shown to follow a negative trend with disease severity meaning a lower frequency in HCC (38%) followed by cirrhosis (47%), CHC with severe fibrosis (51%) and CHC with mild‐moderate fibrosis (52%); chi‐squared test for trend for PD‐1.7 = 5.596, *P* = .018 (Table S5). SVR did not significantly affect the genotype association (Table S6). Patients with a lymphoproliferative disease had a higher frequency of the PD‐1.5 C/C allele (34%) than did patients with CHC (26%); in an overdominant model, OR = 0.60, *P* = .012, *P*‐value remained statistically significant after both Bonferroni and Sidak's corrections.

### 3.2 IFNL4 mutational analysis

**FIGURE 1:**
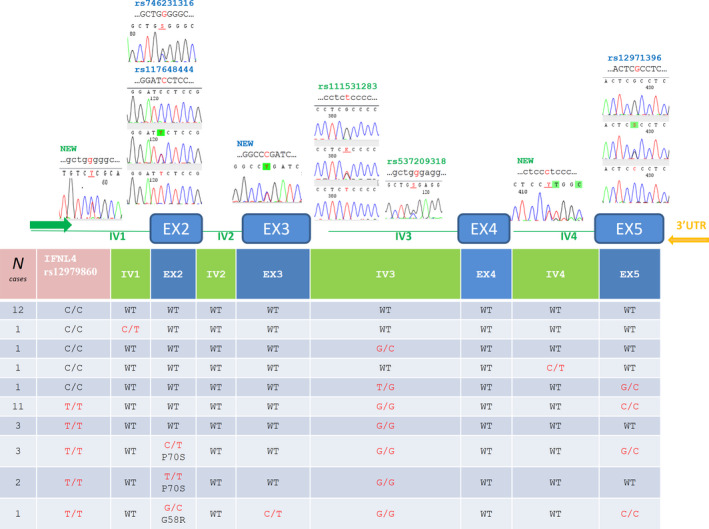
Genomic positions of IFNL4 genetic variants in samples homozygous at rs12979860. In addition to rs12979860, eight different SNPs were identified in 24 of 36 samples. Three SNPs (including one in exon 3) have not previously been reported. The table below the gene diagram indicates the genotype at each polymorphism for all 36 samples

**FIGURE 2:**
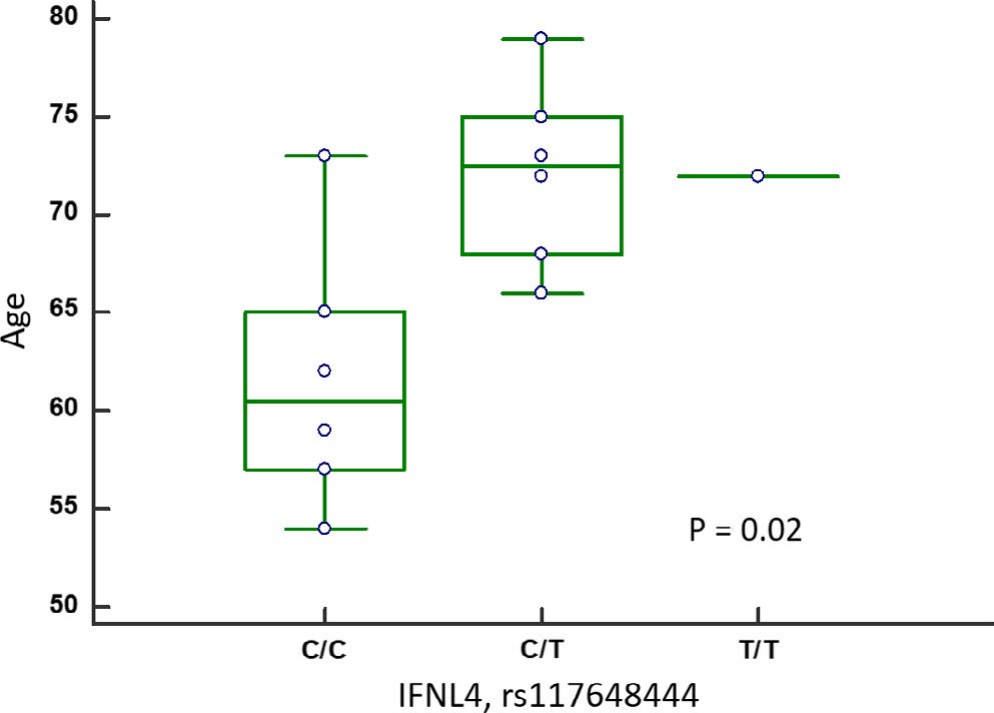
Boxplot describing the relationship of IFNL4 genotype related to a fully active *IFNL4* secretion with a younger median age of HCC patients. HCC patients carrying a fully active (rs12979860‐T/T combined without P70S mutation, rs117648444 C/C) were diagnosed with tumour at younger age compared to those with a reduced expression (rs12979860‐T/T combined with P70S mutation, rs117648444 C/T or T/T), median age 61y and 72y, respectively, *P* = .02 ANOVA test. Boxes range from the 25th to the 75th percentile with a horizontal black line at the median and vertical lines extending to the 10th and 90th percentiles. These data suggest that IFNL4 levels may represent a risk factor for HCC development

The *IFNL4* gene was sequenced from intron 1 to exon 5 in 36 cases homozygous at *IFNL4* rs12979860 with either a C/C (n = 16) or T/T (n = 20) genotype. This analysis identified eight polymorphisms in 24 patients (Figure 1). Already known non‐synonymous variants rs117648444 (P70S, five cases) and rs746231316 (G58R, one case) were identified in exon 2. The amino acids changed by these SNPs flank residue N61, which glycosylation is required for functional IFNλ4.^50^ Consequently, these variants reduce the protein antiviral activity.^51^ Our results showed that these two variants associate with the rs12979860 T/T genotype (six of 20 cases, 30%) only. Finally, a new synonymous mutation was identified in exon 3 (one case), and the known synonymous variant rs12971396 was found in exon 5 (16 cases). This latter SNP associated with the rs12979860 T/T genotype (15 of 20, 75%). Although based on a small number of patients, these data suggest an association between HCC and secretion of fully active IFNλ4 (patients homozygous for rs12979860[T] without a G58R or P70S variant in *IFNL4*; Table 3). Indeed, the rs12979860[T] polymorphism necessary for IFNλ4 production is associated with HCC (Table 2) and the frequency of the genotype necessary for fully active IFNλ4 expression is higher in HCC (no G58R or P70S variant in 15 of 28, 54%) than in all other HCV‐positive patients (17 of 48, 35%) (Table 3). HCC patients with a fully active *IFNL4* expression were diagnosed with tumour at younger age than those with reduced expression (Figure 2). These data suggest that IFNλ4 levels are a risk factor for HCC development.

**TABLE 3:** Frequency of IFNL4 genetic variants associated with IFNλ4 in patients and blood donors

	IFNλ4 expression	
	Fully	Reduced
patients with rs12979860‐TT genotype tested for G58R or P70S IFNL4 variants	G58R or P70S mutation	

		Absent	Present
BD (n = 5)		3 (60%)	2 (40%)
Patients with liver disease (n = 47)	CHC (n = 11) Cirrhosis (n = 8) HCC (n = 28)	5 (45%) 2 (25%) 15 (54%)	6 (55%) 6 (75%) 13 (46%)
Patients with lymphoproliferative disease (n = 29) All HCV‐positive patients excluded HCC (n = 48)	MC (n = 16) NHL (n = 13)	5 (31%) 5 (38%) 17 (35%)	11 (69%) 8 (62%) 31 (65%)

Abbreviations: BD, blood donors; CHC, chronic HCV infection; HCC, hepatocellular carcinoma; MC, autoimmune mixed cryoglobulinaemia; NHL, non‐Hodgkin lymphoma.

### 3.3 PDCD1 polymorphisms and mRNA expression

**FIGURE 3:**
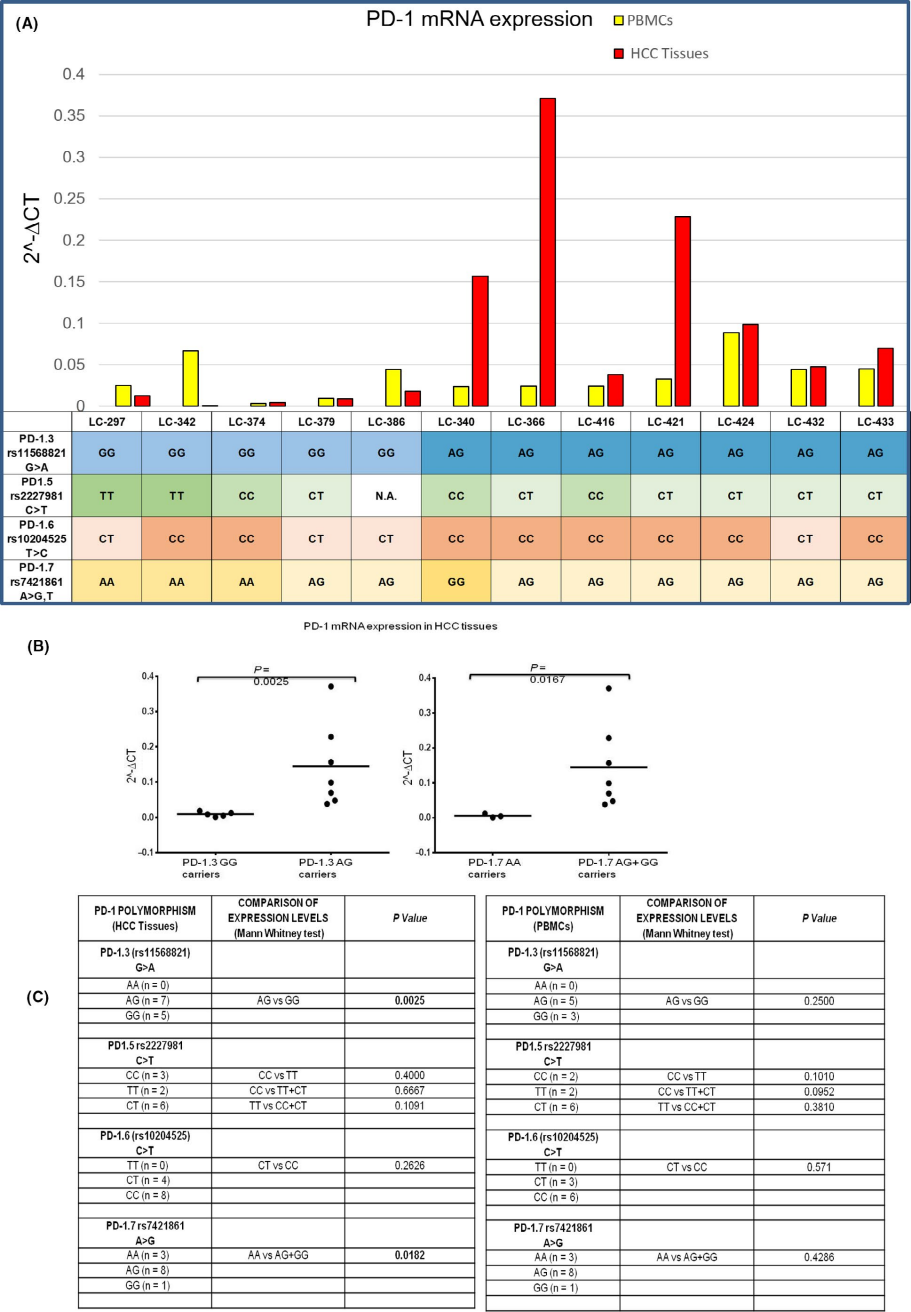
PDCD1 mRNA expression based on PDCD1 haplotype. A, mRNA levels in HCV‐positive HCC biopsy and PBMC from each patients (n = 12) with different PDCD1 polymorphisms. LC‐297 to LC‐433 are the identifier code of patients. Under the column it is reported the haplotype of each single patient. The expression of PDCD1 gene was expressed in the y‐axis as the 2^−ΔCt^ value. The ΔCt values for each amplified transcript were calculated by subtracting the respective Ct value from the corresponding control gene *SRSF4* Ct (ΔCt = Ct_x_ – Ct*_SRSF4_*). B, Median level of PDCD1 mRNA in HCC tissue and PBMC of patients stratified based on PD‐1.3 and PD‐1.7 genotype, Mann‐Witney test. C, Comparison of different expression level of PDCD1 mRNA in HCV‐positive HCC tissue and PBMC from patients based on their PDCD1 genotype (PD‐1.3, PD‐1.5, PD‐1.6. PD‐1.7). The patients having PD‐1.3 allele A and PD‐1.7 allele G exhibited a higher *PD‐1* mRNA expression levels than those with wild‐type homozygote (PD‐1.3 genotype G/G, *P* = .0025 and PD‐1.7 genotype A/A, *P* = .0182, Mann‐Witney test respectively)

We next examined whether *PDCD1* polymorphisms affected mRNA expression in tumour biopsies and PBMCs from HCC patients (Figure 3A). We found higher *PDCD1* mRNA expression in HCC tissues carrying the PD‐1.3 A/G genotype than the G/A genotype (*P* = .0025, Mann‐Whitney test; Figure 3B and C). Similarly, expression was higher in HCC tissues with the PD‐1.7 G polymorphism (A/G and G/G genotypes) than with the A/A genotype (*P* = .0167, Mann‐Whitney test; Figure 3B and C). Conversely, no significant difference in *PDCD1* mRNA expression was observed among PBMCs from HCC patients with different PD‐1 genotypes (Figure 3C). Considering the important role of the PD‐1–PD‐L1 axis in regulating T‐cell function, these results suggest that a high expression of PD‐1 in the HCC microenvironment contributes to hepatocarcinogenesis by locally impairing antitumour immunity.

### 3.4 Epistatic interactions between IFNL4 and PDCD1 variants

**FIGURE 4:**
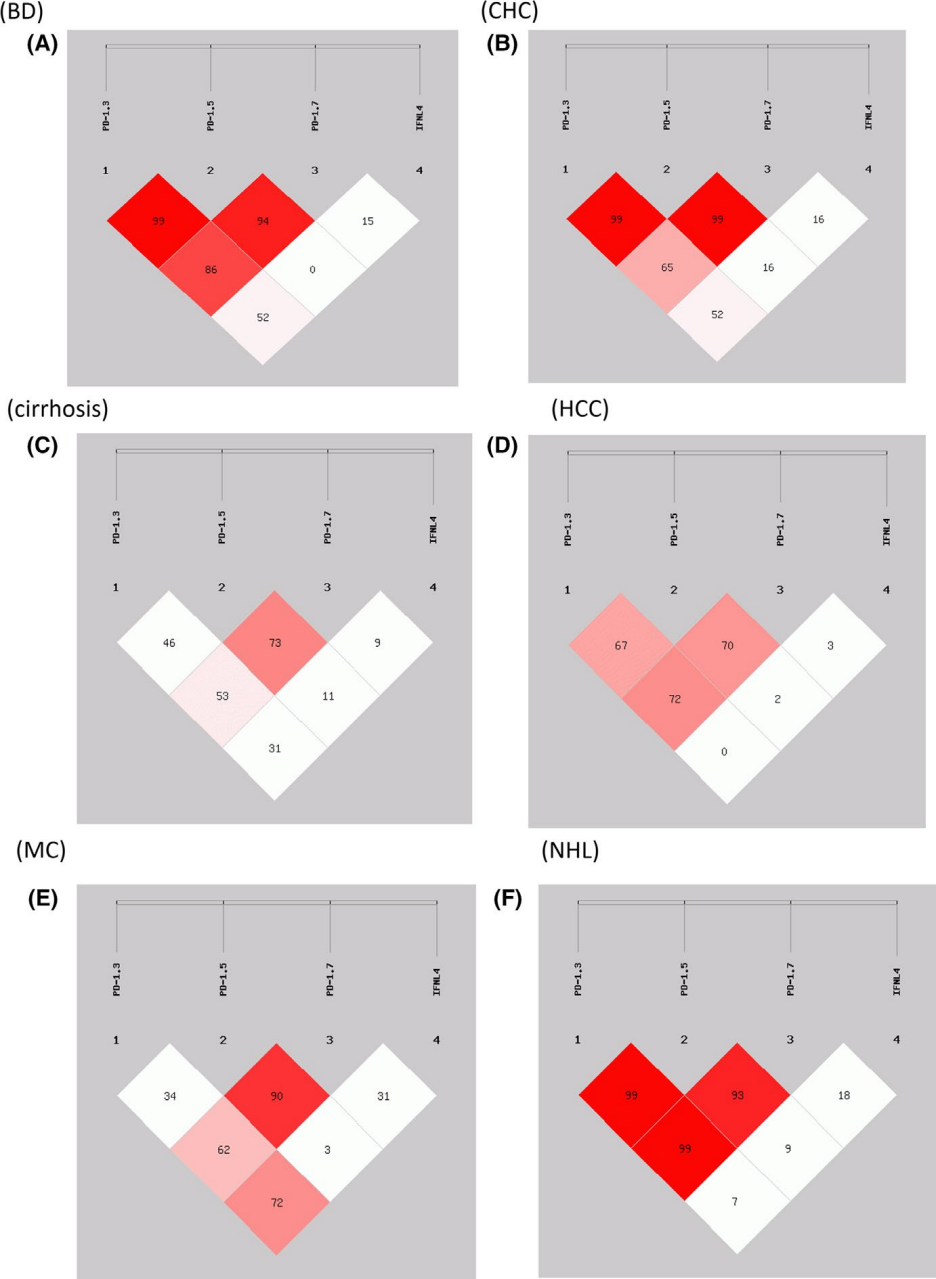
Pairwise linkage disequilibrium (LD) relationships between the *PD‐1.3, PD‐1.5, PD‐1.7 and IFNL4* rs12979860 polymorphism. A–F, Results from linkage analysis conducted for the polymorphism of blood donors (A), CHC (B), cirrhosis (C), HCC (D), MC (E) and NHL (F) respectively

Haplotype data for the *PDCD1* SNPs PD‐1.3, PD‐1.5 and PD‐1.7 and for rs12979860 of *IFNL4* were analysed using SHEsis software, yielding eight common haplotypes with a frequency >5% (Table 4). In addition, a comparison between cirrhosis and HCC has been performed (Table S7). These results suggested that specific haplotypes may influence the susceptibility to or progression of different HCV‐related diseases (Table 4). In addition, linkage disequilibrium analysis (LD) evidenced a linkage that did not occur by chance (LD >50) between *PDCD1* SNPs and rs12979860 of *IFNL4* in BD and in patients with CHC, MC and B‐NHL but not in patients with cirrhosis or HCC. An association between LD‐1.3 and *IFNL4* was found in MC (LD = 72) but not in HCC (LD = 0) (Figure 4). In addition, real‐time polymerase chain reaction (PCR) indicated higher *PDCD1* expression in HCC tissue samples carrying a PD‐1.3 G/A allele and a PD‐1.7 G/A or G/G allele than in those with A/A genotypes, respectively, but not in PBMCs of the same patients (Figure 3).

**TABLE 4:** Epistatic interaction defined by *PD‐1* and *IFNL4* polymorphisms and their associations with HCV‐related diseases compared to patients with a chronic HCV infection

Haplotype				Frequency					Frequency				
PD			IFNL4	Cirrhosis(freq)	CHC(freq)	Χ^2^	*P* value	Odds ratio [95%CI]	HCC(freq)	CHC(freq)	Χ^2^	*P* value	Odds ratio [95%CI]
1.3	1.5	1.7											
A	C	G	C						21.00 (0.053)	8.29 (0.028)	2.82	*0.09*	1.992 [0.878‐4.519]
**A**	**C**	**G**	**T**	3.11 (0.014)	17.50 (0.059)	6.25	***0.012***	**0.237** [0.070‐0.803]	20.51 (0.051)	17.50 (0.059)	0.12	*0.73*	0.890 [0.461‐1.717]
G	C	A	C	27.60 (0.122)	47.60 (0.161)	0.90	*0.343*	0.782 [0.470‐1.301]	56.17 (0.140)	47.60 (0.161)	0.32	*0.57*	0.885 [0.581‐1.348]
**G**	**C**	**A**	**T**	27.32 (0.121)	23.20 (0.078)	3.59	*0.058*	1.753 [0.976‐3.150]	58.56 (0.146)	23.20 (0.078)	8.49	***0.00***	**2.101 [1.265‐3.491]**
G	C	G	C	37.66 (0.167)	33.13 (0.112)	4.56	*0.033*	1.730 [1.042‐2.873]	43.22 (0.108)	33.13 (0.112)	0	*0.99*	0.997 [0.616‐1.613]
G	C	G	T	26.63 (0.118)	25.08 (0.085)	2.31	*0.128*	1.562 [0.876‐2.786]	36.96 (0.092)	25.08 (0.085)	0.23	*0.63*	1.140 [0.670‐1.941]
G	T	A	C	46.53 (0.206)	74.58 (0.252)	0.67	*0.403*	0.835 [0.548‐1.274]	78.78 (0.197)	74.58 (0.252)	2.29	*0.13*	0.756 [0.527‐1.087]
G	T	A	T	30.69 (0.136)	58.42 (0.197)	2.35	*0.126*	0.688 [0.425‐1.112]	61.26 (0.153)	58.42 (0.197)	1.79	*0.18*	0.763 [0.513‐1.135]

PD			IFNL4	MC (freq)	CHC (freq)	Χ^2^	*P* value	Odds ratio [95%CI]	NHL (freq)	CHC (freq)	Χ^2^	*P* value	Odds ratio [95%CI]
1.3	1.5	1.7											
A	C	G	C	29.49 (0.107)	8.29 (0.028)	14.82	***<0.001***	4.237 [1.923‐9.334]					
A	C	G	T	1.71 (0.006)	17.50 (0.059)	12.12	***<0.001***	0.100 [0.021‐0.488]	8.23 (0.030)	17.50 (0.059)	2.67	*0.102285*	0.500 [0.214‐1.165]
G	C	A	C	31.37 (0.114)	47.60 (0.161)	2.45	*0.118*	0.680 [0.418‐1.105]	38.71 (0.143)	47.60 (0.161)	0.33	*0.565905*	0.873 [0.550‐1.387]
G	C	A	T	37.64 (0.136)	23.20 (0.078)	5.38	*0.0208*	1.895 [1.097‐3.275]	27.92 (0.103)	23.20 (0.078)	1.09	*0.296122*	1.360 [0.762‐2.427]
G	C	G	C	41.98 (0.152)	33.13 (0.112)	2.25	*0.134*	1.452 [0.890‐2.370]	36.33 (0.135)	33.13 (0.112)	0.69	*0.407568*	1.237 [0.747‐2.050]
G	C	G	T	25.81 (0.094)	25.08 (0.085)	0.18	*0.669*	1.134 [0.637‐2.019]	18.14 (0.067)	25.08 (0.085)	0.61	*0.432726*	0.778 [0.414‐1.459]
G	T	A	C	68.60 (0.249)	74.58 (0.252)	0	*0.990*	1.003 [0.685‐1.467]	66.75 (0.247)	74.58 (0.252)	0.02	*0.900848*	0.976 [0.664‐1.434]
G	T	A	T	27.60 (0.100)	58.42 (0.197)	10.17	***0.001***	0.458 [0.281‐0.746]	58.62 (0.217)	58.42 (0.197)	0.35	*0.554954*	1.131 [0.751‐1.705]

Note

Multi‐loci genotype frequency with a frequency <0.05 in both control and cases has been dropped. *P* value significant at Bonferroni's correction (*P*‐value threshold of 0.0125) are in bold text and highlighted in red when positive association was found and in grey when a negative association was found.

OR (95% CI), Odds ratio with 95% confidence interval.

CIRRHOSIS:Global chi2 is 18.330076 while *df* = 6; Fisher's *P* value is 0.005516.

HCC: Global chi2 is 13.963398 while *df* = 7; Fisher's *P* value is 0.052061.

MC: Global chi2 is 43.070274 while *df* = 7; Fisher's *P* value is 3.48e‐007.

NHL: Global chi2 is 5.251823 while *df* = 6; Fisher's *P* value is 0.512026.

Abbreviations: BD, blood donors; CHC, chronic hepatitis C virus infection; HCC, hepatocellular carcinoma; hepatic: cirrhosis and HCC; lymph: MC and NHL; MC, autoimmune lymphoproliferative mixed cryoglobulinaemia; NHL, non‐Hodgkin lymphoma.

Overall these results underline the importance of *IFNL4* expression (*IFNL4* rs12979860[T] variant) and low hepatic expression of PD‐1, found in the GCAT haplotype, in predicting HCV‐related HCC (OR = 2.101; 95% CI, 1.265‐3.491, Table 4). Conversely, expression of *PDCD1* (ACGC haplotype) in addition to the more potent anti‐HCV *IFNL3* expression (*IFNL4* rs12979860[C] carrier) was in large part associated with MC (OR = 4.237; 95% CI, 1.923‐9.334). Moreover, when IFNλ4 was expressed (*IFNL4* rs12979860[T] carrier), its antiviral effect in MC patients was frequently reduced by the presence of a G58R or P70S variant in IFNλ4, by lowering the level of IFNλ4 secretion (present in 69% of patients carrying the *IFNL4* rs12979860[T] allele in our series). When IFNλ4 is produced in MC, *PDCD1* expression seems less important (GCAT haplotype; OR = 1.895; 95% CI, 1.097‐3.275). Overall, these data suggest that the PD‐1 SNPs, coupled with a low antiviral effect as a result of a reduced *IFNL4* expression, have an important role in MC while expression of a fully active *IFNL4* (*IFNL4* rs12979860[T] allele without G58R or P70S mutation) is more important in determining hepatic outcomes.

### 3.5 PD‐1 expression on T cells and PD‐L1 expression on B lymphocytes of MC patients

**FIGURE 5:**
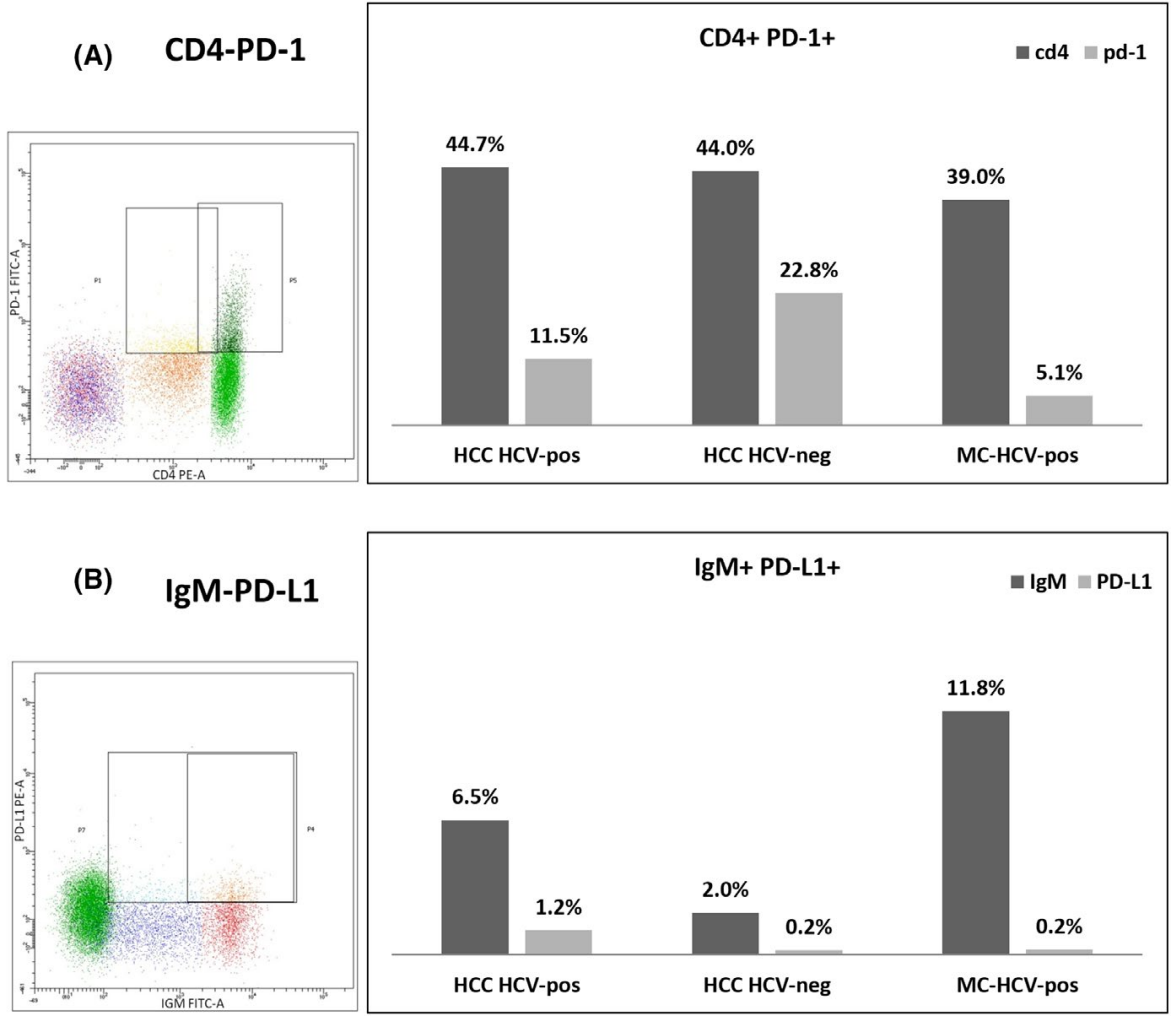
*PD‐1* and *PD‐L1* flow cytometry analysis. The lymphocyte population was selected on a forward‐ versus side‐scatterplot for each patient. PBMCs from HCV‐negative HCC (n = 2), HCV‐associated HCC (n = 2) and HCV‐associated MC cases (n = 2) were analysed by flow cytometry after staining with anti‐CD4 and anti‐PD‐1 antibodies, or anti‐CD19, anti‐IgM and anti‐*PD‐L1* antibodies. Representative dot plot of one case is shown in (A) and (B) respectively

A new B‐cell subpopulation has recently been described, namely CD19^+^ PD‐L1^+^ regulatory B cell, which requires PD‐L1 expression to regulate CD4^+^ PD‐1^+^ T follicular helper (Tfh) cell expansion and differentiation and to suppress autoimmune diseases.^54^ Also in peripheral tissues, and particularly during chronic pathological immune situations, CD4^+^ helper T cells belonging to follicular helper lineage have an important role in stimulating B‐cell responses, including plasma cell differentiation and production of high‐affinity antibodies.^55^, ^56^ Given that PD‐L1^hi^ B cells limit both memory B‐cell development and plasma cell differentiation (pathognomonic signs of MC) by interacting with PD‐1^hi^ T cells,^56^, ^57^ we analysed the expression of PD‐1^hi^ in CD4^+^ T cells and of PD‐L1^hi^ in IgM^+^ CD19^+^ B lymphocytes from patients with HCC without HCV infection (n = 2), with HCV‐related HCC (n = 2) and with HCV‐related MC (n = 2). We observed >10% levels of PD‐1^hi^ expression on CD4^+^ T cells and ≥1% levels of PD‐L1^hi^ on IgM^+^ CD19^+^ B cells in HCC patients without HCV infection (Figure 5A), but lower than 10% levels of PD‐1^hi^ expression on CD4^+^ T cells and absence of PD‐L1 ^hi^ in patients with HCV‐related MC (Figure 5B).

## 4 DISCUSSION

This study confirms the significant associations between the *IFNL4* rs12979860[T] variant and both persistence of HCV infection and risk of HCC.^17^, ^51^, ^58^ The *IFNL4* rs12979860[T] variant is in high LD with the rs3682348[ΔG] polymorphism necessary for producing the antiviral cytokine IFNλ4 while preventing the production of IFNλ3,^51^, ^59^ which has a stronger antiviral activity than IFNλ4. We identified in our series four SNPs in LD with the rs12979860[T] variant: two functional G58R and P70S variants whose minor allele decreased IFNλ4 secretion,^15^ the intronic (IV3) rs111531283[G] variant of unknown role but present in all cases, and the synonymous rs12971396[C] variant in exon 5.^15^ The frequencies of polymorphic alleles encoding a fully active IFNλ4 and a reduced secretion of IFNλ4 (ie G58R and P70S) were not significantly different between HCC patients and BD. Nonetheless, patients with variants leading to a higher *IFNL4* production showed a delay in HCC development (median age, 72 vs 60 years, *P* = .02). A possible explanatory hypothesis is that fully active IFNλ4‐P70 causes a low level of viral replication, which in turn leads to an inefficient adaptive immune response and consequently poor HCV clearance^60^ and increased risk of developing HCC.

PD‐1 inhibitors have been approved for patients with HCC, and several pivotal trials are ongoing.^61^ We found that PD‐1.7 (rs7421861) polymorphism after statistical Bonferroni's correction maintains the significant association with the risk of HCC compared with CHC. Our results suggest that PD‐1 is unrelated to chronic infection because the polymorphic frequency of the same allele is similar in CHC and BD but might influence the clinical outcome of HCV infection. We examined the relation between *PDCD1* polymorphisms and mRNA expression in liver biopsies and PBMCs of selected patients with HCC. Interestingly, HCC biopsies from patients carrying the PD‐1.3 (rs11568821‐A allele) and PD‐1.7 (rs7421861‐G allele) polymorphisms expressed higher levels of *PDCD1* mRNA; this association was not found in PBMCs from the same patients. These results suggest that the HCC microenvironment promotes *PDCD1* expression, whose level could be related to the PD‐1.3 and PD‐1.7 genotypes.

Since it is widely accepted that mutations have different effects in combination than individually, we analysed the relationship between the *PDCD1* haplotype and *IFNL4* in HCC and compared it to that in patients with CHC. The GCAT haplotype, exhibiting lower *PDCD1* mRNA expression in tumour biopsies and a fully active IFNλ4 protein, associated with HCC in our series. Moreover, the ACGC haplotype, exhibiting higher PDCD1 mRNA and a lower IFNλ4 protein expression, discriminates cirrhosis from HCC. Furthermore, LD analysis indicated that PD‐1.3 and PD‐1.7 are in linkage (LD = 72) in HCC and that *IFNL4* is an independent gene (LD = 0). The expression of *PDCD1* in HCC cells is not well characterized. A recent study reported that PD‐1 participates in the mTOR pathway in PD‐1‐positive tumour cells.^62^ However, the specific biological role and target implications of PD‐1‐positive cells in HCC biopsies require further investigation.

The association of *IFNL4*[TT] genotype in patients with HCV‐related lymphoproliferative disorders was less evident than in patients with liver diseases. Furthermore, the association with HCV‐related lymphoproliferative disorders was lower when considering the less active form of IFNλ4 (*IFNL4* rs12979860[T] coupled to P70 mutation), which causes an increase in viral replication.^60^ We hypothesize that HCV replication intensifies the antigen‐driven immune stimulation that in turn sustains B‐cell proliferation.^63^

The LD analysis showed an epistatic contribution between *IFNL4* and PD‐1.3 polymorphisms in MC patients (LD = 72). The study of haplotypes indicated that PD‐1[ACG]‐IFNL4[C] was positively associated with MC (OR = 4.237) and that the opposite haplotype [GTA][T] (OR = 0.458) is negatively associated with MC. These results suggest that that PD‐1.3 G allele may produce a PD‐1 molecule that counterbalances the absence of IFNλ4 protein (rs12979860 = CC) in the risk of MC and vice versa.

Previous studies have demonstrated that high expression of PD‐1 in hepatic lymphocytes, especially exhausted T cells and Tregs, is associated with a dysfunctional immune response in chronic HBV infection and HCC,^64^, ^65^, ^66^, ^67^ and that PD‐1 had influence on the viral profile.^68^ Studies on CHC ^69^, ^70^ showed that overexpression of PD‐1 on HCV‐specific CD8+T cells was associated with a reduced efficiency of T cell–mediated cytolysis compared to non‐HCV–specific T cells, and correlated with the maintenance of an exhausted phenotype of CD8+T cells.^71^ Notably, blockade of PD‐1–PD‐L1 interactions restored the activity of HCV‐specific T cells,^72^ and controlled HCV replication in a chimpanzee model of CHC.^73^

Lymphoid follicles with a germinal centre architecture were commonly observed in the livers of patients with HCV infection.^74^ Recently, a new B‐cell subpopulation, the CD19^+^ PD‐L1^+^ regulatory B cell (Breg), has shown to require PD‐L1 expression to regulate CD4^+^ PD‐1^+^ T follicular helper (Tfh) cell expansion and differentiation and to mediate humoural immunity.^54^ These findings disclosed novel mechanisms by which PD‐1–PD‐L1 signalling regulates antibody production and helps to understand the role of this pathway in physiological states and in the altered humoural immunity found in some autoimmune diseases. Given that PD‐L1^hi^ B cells limit both memory B‐cell development and plasma cell differentiation by interacting with Tfh cells,^56^, ^57^ we analysed the expression of PD‐1^hi^ in CD4^+^ T cells and PD‐L1 ^hi^ in IgM^+^ CD19^+^ B lymphocytes from patients with HCV‐related MC. We observed higher levels (>10%) of PD‐1^hi^ expression on CD4+T cells and lower levels of PD‐L1^hi^ (≤1%) on IgM^+^ CD19^+^ B cells in patients with HCV‐related MC compared to patients with HCC without HCV‐infection, suggesting an alteration in the control of B cell‐T cell interaction in our MC cases. These data are consistent with a model in which the reduction in both CD4+PD‐1^hi^ + T cells and regulatory B cells (PD‐L1^hi^+), necessary to inhibit the signal of terminal plasma cell differentiation and memory B‐cell development, is less effective in MC cases.^54^, ^75^ The favourable actions of CD19^+^ PD‐L1^+^ B cells in humoural B‐cell homeostasis and in the control of autoimmune diseases were further supported by the demonstration that these B cells are resistant to αCD20 B‐cell depletion^54^ and that MC patients also benefit from αCD20 treatment.^76^

This is the first study evaluating a possible association between *PDCD1* polymorphisms and the risk of HCV‐related lymphoproliferative disorders. These results should be considered descriptive, and larger studies are needed to confirm the model of *IFNL4* and *PDCD1* epistatic interactions in HCV‐related MC and the independent contribution of the *IFNL4*[T] variant in the control of HCV infection and HCC development.

Elimination of an infectious agent has been postulated to favour the development of autoreactivity.^77^ Based on our results we proposed that genes encoding immune proteins involved in the control of HCV infection, for example IFNλ4, need genes that encode other inhibitory proteins, for example PD‐1, to inhibit the progression of the disease towards autoimmune MC. The previous study describing that the 15‐year cumulative probability of developing cirrhosis and HCC was higher in MC(−) than in MC(+) patients (24.9% vs 14.2% and 20.3% vs 7.5% respectively),^78^ supports this model. In keeping with these findings, we found different haplotype associations between HCC and MC patients, although further studies across different populations and functional assessments of relevant polymorphisms are required to confirm the associations of pathogenic relevance.

In conclusion, our study found that *IFNL4* and *PDCD1* polymorphisms are both important in determining the risk of HCV‐related MC, although we have yet to determine precisely how the proteins confer this risk. Our data also confirm and underline the important role of INFλ4 production as a risk factor for HCV persistence and HCC development. As a result of the importance of these genes in the immune response to hepatic infection, autoimmune disorders and malignancies, as well as their role in response to the emerging immune checkpoint treatment for HCC, our study adds new information to help understand the pathogenic role of host genetic variants in HCV‐related diseases.

## 5 FUNDING INFORMATION

Mariangela De Zorzi, Laura Caggiari and Ombretta Repetto had fellowships funded by 5X1000_2010_MdS. Francesca Pezzuto is the recipient of a research fellowship awarded by FIRE/AISF ONLUS (Fondazione Italiana per la Ricerca in Epatologia) http://www.fondazionefegato.it/.

## 6 ETHICS STATEMENTS

This study was in accordance with the principles of Declaration of Helsinki and all subjects provided written informed consent. The study protocol was accepted by the Comitato Etico Indipendente of the Azienda Ospedaliero‐Universitaria Consorziale Policlinico di Bari, the Scientific Board and Ethics Committee of Fondazione G. Pascale Istituto Nazionale Tumori, the Comitato Etico Area Vasta Centro AOU Careggi, Florence and Committee for the Bio‐banking Facility of the Centro di Riferimento Oncologico di Aviano.

## CONFLICT OF INTEREST

The authors declare that the research was conducted in the absence of any commercial or financial relationships that could be construed as a potential conflict of interest.

## Supporting information

Table S1

Table S2

Table S3

Table S4

Table S5

Table S6

Table S7
