# Supplementary material for: PDCD1 and IFNL4 genetic variants and risk of developing hepatitis C virus‐related diseases
Source: Liver Int. 2020 Dec 29;41(1):133–49. doi: 10.1111/liv.14667 (PMC7839592; doi:10.1111/liv.14667)
Supplement: Supplementary file 1 — Table S1 [file LIV-41-133-s001.docx]

Supplementary Table 1. Major characteristic of the six PD-1 SNPs reported to be associated with HCV infection Characteristic PD-1.1* PD-1.3 PD-1.5 PD-1.6 PD-1.7 PD-1.9*


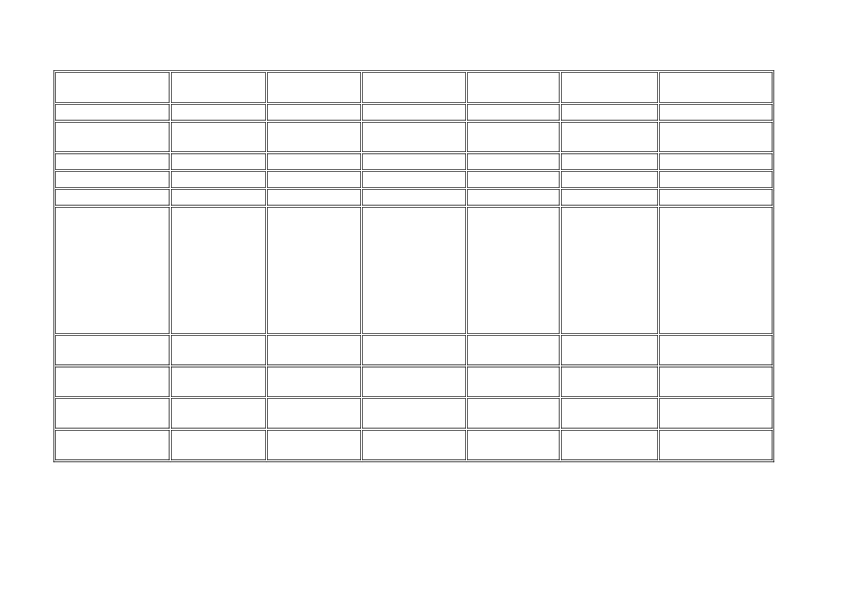


Reference ID rs36084323 G>A rs11568821 G>A rs2227981 C>T rs10204525 C>T rs7421861 A>G rs2227982 A>G Chromosome (2) 242801596 241851760 241851121 241850169 241853198 242793433

Position

Gene position# −606G/A +627+189G/A 804 C/T *889 C/T 77-218 A/G 644 C/T

Region Promoter Intron 4 Exon 5 3′UTR Intron 1 Exon 5

AA substitution alanine→alanine alanine→valine

Effect G>confers A> tandem repeats T> reduces PD-1- T> reduces G> mutation AA substitution in the

increased basal contain binding mediated T cell expression of disrupts the extra-cellular domain, PD-1 expression at sites for inhibition IFN-γ regulatory probably altering the

early-to- transcription elements involved structures and

intermediate factors; increases in the translation of functions of PD-1

stages of CD4+ T- PD-1 expression PD-1, and then

cell activation probably reduces

the expression of

PD-1

MAF for Chinese in 0.490 0.000 0.270 0.302 0.165 0.488

database

MAF for European <0.05 0.122 0.402 0.116 0.352 <0.05

in database

MAF for Toscani in <0.05 0.168 0.421 0.145 0.332 <0.05

Italy population

MAF in healthy <0.05 0.082 0.408 0.097 0.332 <0.05

blood donors

# Gene Reference NM_0050 18.2c.

*PD-1.1 and PD-1.9 were in strict linkage disequilibrium.

PD-1.1 and PD-1.9 had a < 5% MAF in our controls and then were excluded from the subsequent analysis
