# Supplementary material for: PDCD1 and IFNL4 genetic variants and risk of developing hepatitis C virus‐related diseases
Source: Liver Int. 2020 Dec 29;41(1):133–49. doi: 10.1111/liv.14667 (PMC7839592; doi:10.1111/liv.14667)
Supplement: Supplementary file 2 — Table S2 [file LIV-41-133-s002.docx]

Supplementary Table 2. Primer sequences

| IFNL4 FIV1 3UTR | GTAAGTCACCGCCCAGCCCCTGTGCC | Reference 4 |
| --- | --- | --- |
| IFNL4 RIV1 3UTR | CCCATTGACTGAGAGCCTCGCCCGG | Reference 4 |
| IFNL4 int IV1 | GGTTCAGTACACGACAGGCA | Designed by authors |
| PD-1.3_FW | CCAGGCAGCAACCTCAATC | Reference 79 |
| PD-1.3_REV | GTCCCCCTCTGAAATGTCC | Reference 79 |
| PD-1.5_FW | AGACGGAGTATGCCACCATT | Reference 31 |
| PD-1.5_REV | CCAAGAGCAGTGTCCATCCT | Reference 31 |
| PD-1.6_P | GAAGTTTCAGGGAAGGTCAG | Reference 48 |
| PD-1.6_Q | CAGTGTGTGGATGTGAGGAG | Reference 48 |
| PD-1.7_FW | CTCTTAGTAGGAAATCAG | Designed by authors |
| PD-1.7_REV | AATGTCATTGAGAAGTCT | Designed by authors |
| PD-1_RT_For | GAGGGACAATAGGAGCCAGG | Designed by authors |
| PD-1_RT_Rev | TCTTCTCTCGCCACTGGAAA | Designed by authors |
