# Supplementary material for: PDCD1 and IFNL4 genetic variants and risk of developing hepatitis C virus‐related diseases
Source: Liver Int. 2020 Dec 29;41(1):133–49. doi: 10.1111/liv.14667 (PMC7839592; doi:10.1111/liv.14667)
Supplement: Supplementary file 3 — Table S3 [file LIV-41-133-s003.docx]

Supplementary Table 3. Liver fibrosis stage according to PDL-1 and IL-28 genotype and allele distributions in HCV patients with CHC and MC

|  |  |  | **Fibrosis in CHC** | | |  | **Fibrosis in MC** | | | |
| --- | --- | --- | --- | --- | --- | --- | --- | --- | --- | --- |
|  |  | **Mild-moderate**  **n=60** | | **%** | **Advanced**  **n=59** | **%** | **Mild-moderate**  **n=27** | **%** | **Advanced**  **n=24** | **%** |
| **PD-1.3 rs11568821** | **G/G G/A**  **G**  **A** | 46  14  106  14 | | 0.77  0.23  0.88  0.12 | 45  14  104  14 | 0.76  0.24  0.88  0.12 | 18  9  45  9 | 0.67  0.33  0.83  0.17 | 19  5  43  5 | 0.79  0.21  0.90  0.10 |
| **PD-1.5 rs2227981** | **C/C**  **C/T**  **T/T**  **C**  **T** | 16  38  6  70  53 | | 0.27  0.63  0.10  0.58  0.42 | 16  33  10  65  53 | 0.27  0.56  0.17  0.55  0.45 | 8  14  5  30  24 | 0.30  0.52  0.19  0.56  0.44 | 11  8  5  30  18 | 0.46  0.33  0.21  0.63  0.38 |
| **PD-1.6 rs10204525** | **C/C**  **C/T**  **T/T**  **C**  **T** | 51  8  1  110  10 | | 0.85  0.13  0.02  0.92  0.08 | 49  8  2  106  12 | 0.83  0.14  0.03  0.90  0.10 | 21  6  --  48  6 | 0.78  0.22  --  0.89  0.11 | 20  4  --  44  4 | 0.83  0.17  --  0.92  0.08 |
| **PD-1.7 rs7421861** | **A/A A/G G/G**  **A**  **G** | 26  29  5  81  39 | | 0.43  0.48  0.08  0.68  0.32 | 34  20  5  88  30 | 0.57  0.34  0.09  0.75  0.25 | 9  15  3  33  21 | 0.33  0.56  0.11  0.61  0.39 | 10  10  4  30  18 | 0.42  0.42  0.17  0.62  0.38 |
| **IFNL4 rs12979860** | **C/C**  **C/T**  **T/T**  **C**  **T** | 17  38  5  72  48 | | 0.27  0.62  0.08  0.60  0.40 | 17  32  10  66  52 | 0.30  0.55  0.17  0.56  0.44 | 12  12  3  36  18 | 0.44  0.44  0.11  0.67  0.33 | 10  11  3  31  17 | 0.42  0.46  0.12  0.65  0.35 |
