# Supplementary material for: PDCD1 and IFNL4 genetic variants and risk of developing hepatitis C virus‐related diseases
Source: Liver Int. 2020 Dec 29;41(1):133–49. doi: 10.1111/liv.14667 (PMC7839592; doi:10.1111/liv.14667)
Supplement: Supplementary file 4 — Table S4 [file LIV-41-133-s004.docx]

Supplementary Table 4. PDL-1 and IL-28 genotypes and HCV-related diseases in patients who obtained a SVR

|  |  | **CHC** | | | | **Cirrhosis** | | | | **HCC** |  |  |  | **MC** |  |  |  | **NHL** |  |  |  |
| --- | --- | --- | --- | --- | --- | --- | --- | --- | --- | --- | --- | --- | --- | --- | --- | --- | --- | --- | --- | --- | --- |
|  |  | ***SVR***  ***n=71*** | | ***All***  ***n=114*** |  | ***SVR***  ***n=70*** |  | ***All***  ***n=113*** |  | ***SVR***  ***n=31*** |  | ***All***  ***n=200*** |  | ***SVR***  ***n=64*** |  | ***All***  ***n=138*** |  | ***SVR***  ***n=36*** |  | ***All***  ***n=135*** |  |
| **PD-1.3 rs11568821** | **G/G**  **G/A**  **A/A** | 50  22  0 | 0.69  0.31  0 | 114  34  0 | 0.77  0.23  0 | 57  13  0 | 0.81  0.19  0 | 92  21  0 | 0.81  0.19  0 | 19  11  1 | 0.61  0.36  0.03 | 151  45  4 | 0.75  0.23  0.02 | 42  17  0 | 0.71  0.29  0 | 99  39  0 | 0.72  0.28  0 | 30  6  0 | 0.83  0.17  0 | 113  22  0 | 0.84  0.16  0 |
| **PD-1.5 rs2227981** | **C/C**  **C/T**  **T/T** | 26  36  10 | 0.36  0.50  0.14 | 39  85  24 | 0.26  0.57  0.16 | 20  36  14 | 0.29  0.51  0.20 | 34  58  21 | 0.30  0.51  0.19 | 10  16  5 | 0.32  0.52  0.16 | 74  94  32 | 0.37  0.47  0.16 | 25  22  12 | 0.42  0.37  0.20 | 53  62  23 | 0.38  0.45  0.17 | 10  17  9 | 0.27  0.47  0.25 | 41  60  34 | 0.30  0.45  025 |
| **PD-1.6 rs10204525** | **C/C**  **C/T**  **T/T** | 61  10  1 | 0.85  0.14  0.01 | 125  20  3 | 0.84  0.14  0.02 | 55  15  0 | 0.79  0.21  0 | 93  19  1 | 0.82  0.17  0.01 | 24  6  1 | 0.77  0.19  0.03 | 159  39  2 | 0.80  0.20  0.01 | 47  12  0 | 0.80  0.20  0 | 109  28  1 | 0.79  0.20  0.01 | 25  11  0 | 0.70  0.31  0 | 106  27  2 | 0.79  0.20  0.02 |
| **PD-1.7 rs7421861** | **A/A A/G**  **G/G** | 32  31  9 | 0.44  0.43  0.13 | 78  56  14 | 0.53  0.38  0.9 | 37  25  8 | 0.53  0.36  0.11 | 53  44  16 | 0.47  0.39  0.14 | 13  15  3 | 0.42  0.48  0.10 | 75  114  11 | 0.37  0.57  0.06 | 20  32  7 | 0.34  0.54  0.12 | 55  63  20 | 0.40  0.46  0.14 | 16  17  3 | 0.44  0.47  0.08 | 69  54  12 | 0.51  0.40  0.09 |
| **IFNL4 rs12979860** | **C/C**  **C/T**  **T/T** | 24  38  10 | 0.33  0.53  0.14 | 39  87  22 | 0.26  0.59  0.15 | 15  40  15 | 0.21  0.57  0.21 | 26  66  21 | 0.23  0.58  0.19 | 11  15  5 | 0.36  0.48  0.16 | 51  103  46 | 0.26  0.52  0.23 | 21  26  12 | 0.36  0.44  0.20 | 58  61  19 | 0.42  0.44  0.14 | 12  17  7 | 0.33  0.47  0.19 | 45  66  24 | 0.33  0.49  0.18 |
