# Supplementary material for: PDCD1 and IFNL4 genetic variants and risk of developing hepatitis C virus‐related diseases
Source: Liver Int. 2020 Dec 29;41(1):133–49. doi: 10.1111/liv.14667 (PMC7839592; doi:10.1111/liv.14667)
Supplement: Supplementary file 5 — Table S5 [file LIV-41-133-s005.docx]

Supplementary Table 5. Association between PD1C1 and IFNL4 SNPS and increasing severity of liver disease

|  |  | CHC with Mild-moderate fibrosis | | CHC with Advanced fibrosis | | Cirrhosis | | HCC | |  |
| --- | --- | --- | --- | --- | --- | --- | --- | --- | --- | --- |
|  |  | n=60 | % | n=59 | % | n=113 | % | n=200 | % | *P Chi square trend* |
| PD-1.7 rs7421861 | **A/A A/G G/G**  **A**  **G** | 31  26  3  88  42 | 0.52  0.43  0.05  0.68  0.32 | 30  22  7  82  36 | 0.51  0.37  0.12  0.69  0.31 | 53  44  16  150  76 | 0.47  0.39  0.14  0.66  0.34 | **75**  **114**  **11**  264  136 | 0.38  0.57  0.05  0.66  034 | ***0.018***  ***0.007***  *0.54*  *0.56*  *0.56* |
| **IFNL4**  rs12979860 | **C/C**  **C/T**  **T/T**  **C**  **T** | **20**  **37**  **3**  **77**  **43** | 0.33  0.62  0.05  0.64  0.36 | **14**  **34**  **11**  **62**  **56** | 0.24  0.58  0.19  0.53  0.47 | 26  66  21  118  108 | 0.23  0.58  0.19  0.52  0.48 | **51**  **103**  **46**  **205**  **195** | 0.26  0.52  0.23  0.51  0.49 | *0.386*  *0.133*  ***0.004***  *0.034*  *0.034* |

Bonferroni’s and Sidak’s correction for 2 SNPs results in a P-value threshold of 0.025 and of Sidak’s correction of 0.0253, respectively.

Significant trends are in bold text. Abbreviations: CHC, chronic HCV infection; HCC, hepatocellular carcinoma
