# Supplementary material for: PDCD1 and IFNL4 genetic variants and risk of developing hepatitis C virus‐related diseases
Source: Liver Int. 2020 Dec 29;41(1):133–49. doi: 10.1111/liv.14667 (PMC7839592; doi:10.1111/liv.14667)
Supplement: Supplementary file 6 — Table S6 [file LIV-41-133-s006.docx]

Supplementary Table 6. PD1C1 and IFNL4 SNPS frequency in patients with sustained virological response

|  |  | CHC  from SVR | | CHC  no/incomplete  responder | | | Cirrhosis  from SVR | | Cirrhosis  no/incomplete  responder | | HCC  from SVR | | HCC no/incomplete  responder | |
| --- | --- | --- | --- | --- | --- | --- | --- | --- | --- | --- | --- | --- | --- | --- |
|  |  | n=70 | % | | n=30 | % | n=70 | % | n=12 | % | n=31 | % | n=3 | % |
| D-1.7  rs7421861 | **A/A**  **A/G**  **G/G**  **A**  **G** | 32  31  9  95  49 | 0.44  0.43  0.13  0.68  0.32 | | 16  12  2  44  16 | 0.53  0.40  0.07  0.69  0.31 | 37  25  8  99  41 | 0.53  0.36  0.11  0.66  0.34 | 5  5  2  15  9 | 0.42  0.42  0.17  0.62  0.38 | 13  15  3  41  21 | 0.42  0.48  0.10  0.66  034 | 0 0.00  3 100  0 0.00  3 0.50  3 0.50 | |
| **IFNL4**  rs12979860 | **C/C**  **C/T**  **T/T**  **C**  **T** | **24**  **38**  **10**  **86**  **58** | 0.33  0.53  0.14  0.61  0.39 | | **7**  **21**  **2**  **35**  **25** | 0.23  0.70  0.07  0.58  0.42 | 15  40  15  70  55 | 0.21  0.57  0.21  0.50  0.50 | **3**  **7**  **2**  **13**  **11** | **0.25**  **0.58**  **0.17**  **0.54**  **0.46** | **11**  **15**  **5**  **37**  **25** | 0.36  0.48  0.16  0.60  0.40 | 1 0.33  2 0.66  0 0.00  4 0.067  2 0.033 | |

Abbreviations: CHC, chronic HCV infection; HCC, hepatocellular carcinoma
