# Supplementary material for: PDCD1 and IFNL4 genetic variants and risk of developing hepatitis C virus‐related diseases
Source: Liver Int. 2020 Dec 29;41(1):133–49. doi: 10.1111/liv.14667 (PMC7839592; doi:10.1111/liv.14667)
Supplement: Supplementary file 7 — Table S7 [file LIV-41-133-s007.docx]

**Supplementary Table 7. Epistatic interaction defined by *PD-1* and *IFNL4* polymorphisms and their associations with cirrhosis compared to patients with HCC**

| **HAPLOTYPE** | | | | **FREQUENCY** | |  |  |  |
| --- | --- | --- | --- | --- | --- | --- | --- | --- |
| **PD** | | | **IFNL4** | **CIRROSIS(freq)** | **HCC(freq)** | **Χ^2^** | ***P* value** | **Odds Ratio [95%CI]** |
| **1.3** | **1.5** | **1.7** |  |  |  |  |  |  |
| G | T | A | C | 46.53(0.206) | 78.78(0.197) | 1.20 | *0.270* | 1.263 [0.832-1.918] |
| G | C | G | C | 37.66(0.167) | 43.22(0.108) | 1.95 | *0.162* | 1.388 [0.875-2.198] |
| **A** | **C** | **G** | **C** | 29.49(0.107) | 8.29(0.028) | 14.82 | ***<0.001*** | 4.237 [1.923-9.334] |
| G | C | A | C | 27.60(0.122) | 56.17(0.140) | 0.24 | *0.630* | 0.885 [0.541-1.449] |
| G | C | A | T | 27.32(0.121) | 58.56(0.146) | 2.97 | *0.085* | 0.658 [0.407-1.062] |
| G | C | G | T | 26.63(0.118) | 25.08(0.085) | 2.31 | *0.128* | 1.562 [0.876-2.786] |
| G | T | A | T | 27.60(0.100) | 58.38(0.146) | 0.158 | *0.691* | 0.909 [0.566-1.458] |
| A | C | G | T | 3.11(0.014) | 20.51(0.051) | 4.92 | *0.027* | 0.276 [0.082-0.928] |

Multi-loci genotype frequency with a frequency <0.05 in both control and cases has been droped.

P value significant at Bonferroni’s correction (P-value threshold of 0.0016) are in bold text .

OR (95% CI), Odds ratio with 95% confidence interval.

Global chi2 is 36.450783 while df=9;Fisher's p value is 0.0000344, after Bonferroni’s correction remains significant (<0.0016)

Abbreviations: BD, blood donors; CHC, chronic hepatitis C virus infection; HCC, hepatocellular carcinoma; MC, autoimmune lymphoproliferative mixed cryoglobulinemia; NHL, non-Hodgkin lymphoma; hepatic: cirrhosis and HCC; lymph: MC and NHL.
